# Differentially expressed proteins in the skin mucus of Atlantic cod (*Gadus morhua*) upon natural infection with *Vibrio anguillarum*

**DOI:** 10.1186/1746-6148-9-103

**Published:** 2013-05-14

**Authors:** Binoy Rajan, Jep Lokesh, Viswanath Kiron, Monica F Brinchmann

**Affiliations:** 1 Faculty of Biosciences and Aquaculture, University of Nordland, Bodø 8049, Norway

**Keywords:** Comparative proteome, Skin mucosa, Atlantic cod, *Vibrio anguillarum*, Bath challenge

## Abstract

**Background:**

Vibriosis caused by *V. anguillarum* is a commonly encountered disease in Atlantic cod farms and several studies indicate that the initiation of infection occurs after the attachment of the pathogen to the mucosal surfaces (gut, skin and gills) of fish. Therefore it is necessary to investigate the role of different mucosal components in fish upon *V. anguillarum* infection. The present study has two parts; in the first part we analyzed the differential expression of skin mucus proteins from Atlantic cod naturally infected with *V. anguillarum* using two dimensional gel electrophoresis coupled with mass spectrometry. In the second part, a separate bath challenge experiment with *V. anguillarum* was conducted to assess the mRNA levels of the genes in skin tissue, corresponding to the selected proteins identified in the first part.

**Results:**

Comparative proteome analysis of skin mucus of cod upon natural infection with *V. anguillarum* revealed key immune relevant proteins like calpain small subunit 1, glutathione-S-transferase omega 1, proteasome 26S subunit, 14-kDa apolipoprotein, beta 2-tubulin, cold inducible RNA binding protein, malate dehydrogenase 2 (mitochondrial) and type II keratin that exhibited significant differential expression. Additionally a number of protein spots which showed large variability amongst individual fish were also identified. Some of the proteins identified were mapped to the immunologically relevant JNK (c-Jun N-terminal kinases) signalling pathway that is connected to cellular events associated with pathogenesis. A bath challenge experiment with *V. anguillarum* showed differential expression of beta 2-tubulin, calpain small subunit 1, cold inducible RNA binding protein, flotillin1, and glutathione S-transferase omega 1 transcripts in the skin tissue of cod during early stages of infection.

**Conclusions:**

Differentially expressed proteins identified in the cod skin mucus point towards their possible involvement in *V. anguillarum* pathogenesis. The role of some of these proteins in vibriosis in cod described in this paper can be considered unconventional with respect to their established functions in higher vertebrates. Based on the differential expression of these proteins they are possibly important components of fish defence against bacteria and innate immunity at large. The feasibility of utilizing these proteins/genes as markers of bacterial infection or stress in cod needs to be explored further.

## Background

Fish like Atlantic cod, of the gadoid lineage, are thought to rely extensively on innate immune components [1,2] to ward off infection. Skin mucus is considered to be one of the foremost barriers against pathogen invasion and several mucosal components like lectins, proteases, mucins etc. have been shown to be relevant in the survival of fish [3].

*Vibrio anguillarum* strains O2α and O2β are commonly associated with vibriosis in cod [4,5] and these infections can cause bleeding skin lesions/ulcers and septicemia, resulting in mass mortalities of farmed cod [6]. The ubiquitous distribution of *V. anguillarum* in various marine habitats, their opportunistic nature, and the sporadic recurrences of vibriosis caused by deviating serotypes make the complete eradication of this disease in fish farms infeasible [7,8].

Vibrio infection in mammals is initiated at gut mucosal surfaces [9]. This information spurred investigations on the role of mucosal surfaces in fish, especially in the gut during the progress of vibriosis. Early studies on carp, anally intubated with *V. anguillarum* bacterins, have shown antigen uptake in the second gut segment and subsequent detection of specific antibodies in the gut mucus, skin mucus and serum, indicating a common mucosal response to the antigen [10]. Other studies have shown that establishment of bacteria in the gut is facilitated by chemotaxis, induced by mucosal components such as amino acids, carbohydrate moieties on proteins like high molecular weight mucins, and bile components [11]. Skin is also considered as a potential infection route for *V. anguillarum*, besides gills and gut [7]. Bath challenge experiments have shown that the primary site of attachment and colonization of *V. anguillarum* is the skin and intestine of zebrafish (*Danio rerio*) [12], and skin and fins of juvenile rainbow trout [13]. *V. anguillarum* virulence is associated with extracellular components like proteases which can cause localized inflammation at the skin surface [6] and it has been shown that epithelial cells in rainbow trout skin can phagocytose *V. anguillarum*[14]. Further, mucosal antibody production has been quantified upon *V. anguillarum* infection [15]. These findings indicate that the pathogen invasion can trigger immune responses in skin and its associated mucosal surface.

Modern techniques like proteomics and transcriptomics can be made use of to study the proteins and genes in the skin mucosa of Atlantic cod afflicted with vibriosis. Such approaches would help us understand host inflammatory responses during disease progression and may pave way for the discovery of vibriosis-related biomarkers. Therefore in the present study we used comparative proteomics to identify differentially expressed proteins from the skin mucus of cod during a natural outbreak of vibriosis. Further, following a *V. anguillarum* challenge, we examined the transcriptional profiles of the genes, corresponding to the selected proteins, in the skin of cod.

## Methods

### *V. anguillarum* infected Atlantic cod juveniles

Atlantic cod juveniles hatched (eggs obtained from Cod Farmers ASA, Norway) at Mørkvedbukta Research Station, University of Nordland, Bodø, Norway in early spring 2011 had a natural outbreak of vibriosis during late summer 2011. These were non-vaccinated fish of size 35-50 g, maintained at 7°C, and fed commercial juvenile feed (Amber Neptun) from Skretting (Stavanger, Norway). Fish from 3 tanks that were diagnosed with vibriosis by Norwegian Veterinary Institute, North-Norway (Harstad; primary agency for aquaculture disease diagnosis in northern Norway) were used for the first study. The fish in these tanks had experienced stress due to handling of fish while grading and a rise in water temperature (7°C to 10°C). The cumulative mortalities recorded over a period of 15 days in the three tanks were 28, 41 and 52%. The fish samples were collected at the end of the recorded mortality period of 15 days starting from first mortality. These fish appeared to be moribund and had bloodshot fins with faint hemorrhages around the fin base. Six fish that were randomly sampled from these tanks to obtain skin mucus were infected with *V. anguillarum*, as confirmed by identifying the bacteria grown on TSA-blood agar with salt [16]. Mucus from six fish of the same stock maintained in separate tanks in a different fish hall, which were confirmed to be not infected, was also sampled. There were no mortalities observed in these control fish group over the same period.

### Mucus sample

Six fish each from infected and non-infected groups were anesthetized in MS-222 and killed by a blow to the head for sampling. Mucus collection was done from normal skin surface avoiding collection of blood along with mucus. Mucus was scraped off with sterile micro slides, transferred into an eppendorf tube and were immediately frozen at 80°C. All the protocols employed in this study were in accordance with the guidelines of Norwegian Animal Research Authority (FDU, http://www.fdu.no/fdu/).

### Sample preparation and two dimensional gel electrophoresis

Skin mucus samples were directly solubilized in sample buffer (Bio-Rad, CA, USA) containing 7 M urea, 2 M thiourea, 1% (w/v) ASB-14, 40 mM Tris, 0.001% Bromophenol Blue modified with 0.5% Bio-Lyte 3/10 ampholyte (Bio-Rad) and 50 mM DTT, vortexed for 15 min at room temperature (RT) and centrifuged at 20000 × *g* for 30 min. The supernatant was transferred to a fresh tube and kept on ice. An aliquot of the supernatant solution was dialysed using nanosep omega 3 kDa cut off columns (Pierce, IL, USA) and the protein quantity in the original solution was measured using Qubit protein assay kit (Invitrogen, Paisley, UK). Hundred μg protein sample was rehydrated in 17 cm 3–10 IPG strips (Bio-Rad) and isoelectric focusing (IEF) was carried out using protean IEF cell (Bio-Rad). After IEF, the strips were immersed in equilibration buffer (6 M urea, 0.375 M Tris–HCl, pH 8.8, 2% SDS, 20% glycerol) containing 0.2% DTT and again in the same buffer containing 0.25% iodoacetamide. The equilibrated strips were loaded on to 12% SDS-PAGE gels and run on PROTEAN IIxi system (Bio-Rad). The gels were stained overnight with SYPRO Ruby (Invitrogen) according to supplier’s protocol and imaging and documentation was carried out using ChemiDoc™ XRS imaging system (Bio-Rad). Gel images were analysed using PD Quest Basic software (Bio-Rad). The gel image analysis workflow included spot detection, spot normalization using local regression mode available in the software and finally spot matching and differential expression analysis. Datasets were generated for spots showing significant differential expression using two-tailed Student’s t-test for means with 95% confidence level and *p* <0.05. A fold change threshold of 1.5 was also set to generate a separate dataset. The density values for the spots were then exported to Microsoft Excel (Microsoft, WA, USA) to recalculate the means and find the exact *p* values as well as creating bar charts.

### LC-MSMS analysis

Eight protein spots showing significant differences and 19 spots that were showing –differential expression trend were excised from preparative SYPRO Ruby stained gels loaded with 500 μg protein. The protein spots were in-gel reduced, alkylated and trypsinised as described elsewhere [17] and subjected to LC-MSMS analysis (ESI Quad TOF; Micromass/Waters, MA, USA). Peak lists (pkl) generated by the Protein Lynx Global server software (version 2.1, Micromass/Waters, MA, USA) were adjusted with an internal trypsin standard and further employed for protein database searching.

### Bioinformatic analysis

The peak list files obtained from LC-MSMS analyses were submitted to MASCOT [http://www.matrixscience.com] search engine and searched against NCBInr with the following parameters: maximum one missed cleavage by trypsin, peptide mass tolerance 100 ppm, MS/MS ion tolerance set to 0.1 Da, fixed carbamidomethyl modification and variable oxidation modification. Protein hits not satisfying a minimum score of 40 and with a low sequence coverage were further searched against vertebrate EST database, taxonomy Actinopterygii. An interaction network of selected proteins was generated based on their mammalian orthologues (generated through BLASTp searches in NCBI database) using Ingenuity pathway analysis [http://www.ingenuity.com].

### Study on experimentally infected fish

The bacterial challenge experiment was conducted at the Fish Health Laboratory of the Institute of Marine Research (IMR), Bergen, Norway in cooperation with University of Nordland (UiN), Bodø, Norway. The study was approved by Norwegian Animal Research Authority (FDU; FDU case number 09/112472, approved 22.09.2009) and protocols employed were in accordance with their guidelines. The fish used were juvenile, non vaccinated Atlantic cod procured at 30 g size (0-yr class) from a commercial hatchery. Prior to the challenge study, groups of 24 fish each of average weight 90 g were introduced into two 500 L experimental tanks. The fish were fed on a laboratory prepared fishmeal-based feed with the nutrient profiles similar to that of commercial formulations. For performing the bacterial challenge, the water flow into the tanks was stopped for a period of one hour. The fish were then either bath challenged with *Vibrio anguillarum* (strain H610 from the collection of the Fish Health Group at IMR) or mock challenged with culture media (tryptic soy broth with 1.5% NaCl). The bacterial amount employed for the challenge was 1.6x10^7^cfu ml^-1^. Skin and gill samples were collected at 0 h (4 fish, initial sample) and from both treatment groups (5 fish from each group) at 4 h and 48 h post challenge and were immediately snap frozen in liquid nitrogen.

### RNA extraction and cDNA synthesis

RNA was extracted from cod skin and gills using Trizol method as described elsewhere [18]. Total RNA was quality checked on 1% agarose gels, quantified using Qubit RNA assay (Invitrogen) and treated with gDNA wipe out buffer. cDNA was synthesized from 1 μg RNA using QuantiTect Reverse Transcription kit (Qiagen, Hilden, Germany).

### Primer design and qPCR

A tool box of genes identified from the proteome experiment were employed for quantitative real-time PCR. Primers were designed using Primer3 software available online [http://frodo.wi.mit.edu/], after retrieving their full length or partial sequences from Atlantic cod genome database [http://www.ensembl.org] or NCBI dbEST and they were then evaluated using Netprimer (PREMIER Biosoft, USA). The oligonucleotide primers and their attributes are shown in Table 1. Novel full/partial EST sequences were cloned by TA Topo cloning kit (Invitrogen) and sequenced as previously reported [18].

**Table 1:** Oligonucleotide primers employed in this study

**Gene name**		**Sequence (5’-3’)**	**Primer**	**Purpose**	**Acc.**
			**efficiency (%)**		**number**
Profilin2 (*Pfn2*)	F^1^	CCAGCCACTCAATATGTCGT		cloning	[GenBank:KC460541]
	R^2^	CAAGAACCTGGGAAACACAC			
	F	ATCGCAGCACTTTATTCACC	95.18	qPCR	
	R	GCGTCCTCCTTGTAGAGGTT			
Peroxiredoxin 6 (*Prdx6*)	F	TATCGGTGAGACAGGACCAT		cloning	[GenBank:KC460539]
	R	TGGCGGTTTATTCTAAGTGC			
	F	GATGAGATCGACAAGGATGG	96.99	qPCR	
	R	TCTATGACCCTCAGCAGCTC			
Flotillin1 (*Flot1*)	F	TCGCTGAAATAGGTCTCTGG		cloning	[GenBank:KC460542]
	R	ACTCAGCCTTCTTGGTGTTG			
	F	ACCAGGACTACCTCCACTCC	91.88	qPCR	
	R	TACTGGGCAGACACCTTCTC			
Calpain small subunit 1 (*Capns1*)	F	TTCTCTTCTCACCGCAGAAC		cloning	[GenBank:KC460540]
	R	GACTTTTCCAGCTCCTCCTC			
	F	AACATGGCAACATGGACTTT	94.05	qPCR	
	R	TTGACATCAAGGGAGATGGT			
Beta 2 Tubulin (*Tubb2*)	F	CAGCTACTTCGTGGAATGGA	94.47	qPCR	[GenBank:AAD56401]
	R	CTGTTGCCGATGAAGGTTAC			
Cold inducible RNA binding protein (*Cirbp*)	F	CTCTTCGGAACTCTCAACCA		cloning	[GenBank:KC460543]
	R	AATACAGCCAGCCAAGTGAC			
	F	GTATGGAAACATCGCCAAAG	94.17	qPCR	
	R	CTCGTCGGCATTATCAAACT			
Glutathione S-transferase omega1(*Gsto1*)	F	ATTGCGTCTTGGTCATTCAT		cloning	[GenBank:KC460544]
	R	ACAGGCCATAGTCGTAGTCG			
	F	CTGAAACACTTCCTGGATGG	95.60	qPCR	
	R	GCGTGCATGGTTTCTTTAAC			
Ubiquitin (*Ubi*)	F	GGCCGCAAAGATGCAGAT	96.10	qPCR	[GenBank:EX735613]
	R	CTGGGCTCGACCTCAAGAGT			
Acidic ribosomal protein (*Arp*)	F	TGATCCTCCACGACGATGAG	95.37	qPCR	[GenBank:EX741373]
	R	CAGGGCCTTGGCGAAGA			
Interleukin 1β (*il1β*)	F	GGAGAACACGGACGACCTGA		qPCR	[GenBank:AJ535730]
	R	CGCACCATGTCACTGTCCTT			

^1^Forward primer.

^2 ^Reverse primer.

qPCR was performed with StepOne thermocycler (Applied Biosystems (AB), CA, USA). A 10 μL PCR mix per reaction was prepared with 1 μL of primers (5 pM each), 5 μL of Fast SYBR Green Master Mix (AB) and 4 μL of 25x diluted cDNA template. The reaction mix was dispensed into 96 well plates and covered with adhesive optical film (AB) and amplification was performed according to the following conditions: holding stage for 20 sec at 95°C for activation of fast Taq DNA polymerase followed by 40 cycles of denaturation at 95°C for 3 sec and annealing/extension for 30 sec at 60°C. Fluorescence data was acquired during the annealing/extension step. The samples were run in duplicates and pooled DNase treated RNA and no template negative controls were also included in the scheme. A 3-fold dilution series from pooled cDNA were included in each plate for each gene and efficiency (E) of PCR reaction for each set of primers was calculated based on standard curve method according to the formula E = 10^(-1/slope)^-1*100 . The Ct values obtained for each gene was transformed into relative quantities by setting the highest value to 1 according to the requirement of geNORM software [http://medgen.ugent.be/~jvdesomp/genorm/] to obtain the normalization values for each sample calculated based on the geometric mean of reference genes ubiquitin (*Ubi*) and acidic ribosomal protein (*Arp*). The normalized values of individual samples were used for statistical analysis.

### Statistical analysis

The statistical analyses of qPCR data were carried out using GraphPad Prism software (GraphPad Software, Inc. CA, USA). The sample sets were tested for normality using Kolmogorov-Smirnov test and data was log transformed where necessary. Transcript profiles for each gene from bacterial challenge group were compared with the respective values from mock challenge control groups, at each time point, using unpaired student’s t-test. Zero hour samples that are included in the bar charts as a basal reference point were not included for statistical comparisons.

## Results & discussion

### Proteomic studies on skin mucus from naturally infected fish

**Figure 1:**
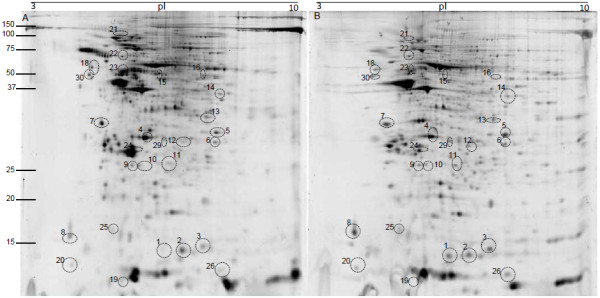
**Representative 2D gels of Atlantic cod skin mucus A) Healthy and B) *V. anguillarum *infected. **Annotated spots were identified using LC-MSMS. The mucus proteins were isoelectrically focused on 17cm IPG strips (pI 3–10) in first dimension, run on a 12.5% SDS-PAGE in second dimension and stained with SYPRO Ruby fluorescent dye. Note that the spot numbers correspond to the protein identities outlined in Table 2. N = 6.

**Figure 2:**
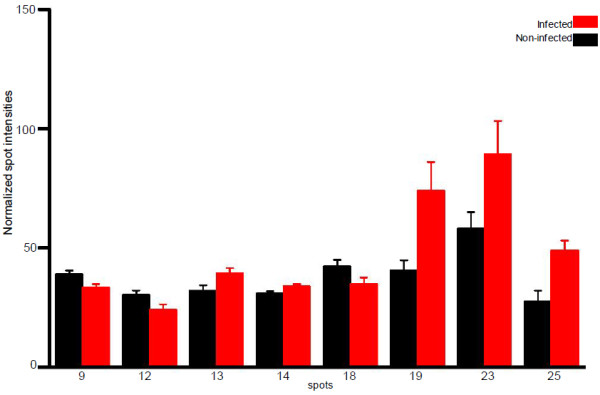
**Normalized spot intensities of protein showing statistically significant differences (p < 0.05) in healthy *vs V. anguillarum *infected cod skin mucus. **Data based on comparative 2D gel analysis employing PD Quest Basic software. N = 6.

Skin mucus is a critical component of piscine immune system. Various antibacterial and inflammatory factors like immunoglobulin, lectins, acute phase proteins, and proteases have been identified in fish mucus [19-21]. We have earlier reported a proteome reference map of Atlantic cod skin mucus, identifying among others innate immune components such as lysozyme, apolipoproteins, galectin-1 and serpins [22]. A separate study showing the presence of antimicrobial components in different tissues including skin mucosa of cod is also available [23]. These studies indicate that the cod skin mucus harbours a significant number of immune relevant proteins. In the present study the skin mucus proteome of healthy cod was compared with that of *V. anguillarum* infected cod. The skin mucus 2D gels showed good resolution and reproducibility (Figure 1). Through gel image analysis, only 8 protein spots were found to be differentially expressed (Figure 2). An additional 19 spots were identified based on high changes observed in individual infected fish (based on variation in spot intensities). There is however, no statistical support for their identification as indicated in Table 2. Nevertheless, this information could be used for further studies, especially targeted transcriptomics or proteomics under natural and bath infection scenarios in fish. The spot details along with accession numbers and other parameters are shown in Table 2.

**Table 2:** Proteins identified in Atlantic cod skin mucus

**N**	**Protein name (Species)**	**Acc. no.**	**Theoretical pI/MW**	**IS**	**SC (%)**	**Cod EST**	**FCx`**	***p***
						**Acc. no**		
1	Profilin-2 (*Danio rerio*)	NP_958493	6.09/14.6	474	35	GO379352	<1.5	>0.05
2	**Profilin-2 ( *Salmo salar *)**	**ACI66182**	**6.72/14.5**	**393**	**33**	**FG312066**	**1.6**	**>0.05**
3	Nucleoside diphosphate kinase (*G. morhua*)	AET05826	7.84/15.5	623	77	NA	<1.5	>0.05
4	Inositol monophosphatase 1(*Anguilla anguilla*)	CBI68709	5.35/30.6	699	35	GW854257	<1.5	>0.05
5	Guanine nucleotide binding protein polypeptide 2 like-1 (*S. salar*)	ACH70647	7.64/28.6	675	44	GW858107	<1.5	>0.05
6	Voltage dependent anion selective channel protein 1 (*S. salar*)	ACI33832	8.69/30.9	664	27	GW858279	<1.5	>0.05
7	Tropomyosin-alpha-4 chain (*S. salar*)	ACN10541	4.57/27.9	760	39	FF409290	<1.5	>0.05
8	Calmodulin (*S. salar*)	ACI68592	4.09/16.7	103	19	NA	<1.5	>0.05
9	**Calpain small subunit-1 ( *S. salar *)**	**ACI167026**	**5.34/24.6**	**428**	**44**	**EX730492**	**<1.5**	**0.0233**
10	Calpain small subunit-1 (*S. salar*)	ACI167026	5.34/24.6	731	42	GW858828	<1.5	>0.05
11	Peroxiredoxin-6 (*Scophthalmus maximus*)	ADJ57694	5.46/24.3	306	44	ES239904	<1.5	>0.05
12	**PREDICTED: Glutathione S-transferase omega-1 like ( *Oreochromis niloticus *)**	**XP_003438319**	**6.01/27.4**	**201**	**22**	**FCO73610**	**<1.5**	**0.0409**
13	**Malate dehydrogenase 2-NAD (mitochondrial) ( *S. salar *)**	**NP_001133198**	**8.15/35.8**	**327**	**19**	**NA**	**1.52**	**0.0221**
14	**Proteasome (macropain) 26S subunit ( *S. salar*)**	**ACH85257**	**6.81/44.4**	**246**	**19**	**NA**	**<1.5**	**0.0130**
15	PREDICTED: alpha-enolase-like isoform 1 (*S. salar*)	XP_003444858	5.99/47.5	791	46	NA	<1.5	>0.05
16	Flotillin-1 (*Larimichthys crocea*)	AEP68103		295	17	NA	<1.5	>0.05
18	**Type II keratin E3 ( *Oncorhynchus mykiss *)**	**NP_001123458**	**5.32/55.3**	**380**	**20**	**GW854186**	<1.5	**0.0413**
19	**14kDa apolipoprotein ( *Epinephelus coioides *)**	**ACM41842**	**6.26/15.4**	**249**	**35**	**EX189720**	**3.6**	**0.0238**
20	PREDICTED: similar to ribosomal protein P2 (*Taeniopygia guttata*)	XP_002199373	4.21/11.7	327	57	EX174193	<1.5	>0.05
21	PREDICTED: endoplasmin-like (*O. niloticus*)	XP_003443932	4.75/91.6	339	24	GW849407	<1.5	>0.05
22	**Serine/threonine-protein phosphatase2A 65 kDa regulatory subunit A beta isoform ( *S. salar *)**	**ACN58704**	**4.94/65.9**	**355**	**29**	**NA**	**2.3**	**>0.05**
23	**Beta-2-tubulin ( *G. morhua *)**	**AAD56401**	**4.71/50.1**	**670**	**36**	**NA**	**2.5**	**0.0419**
24	**Preproapolipoprotein A-1 ( *G. morhua *)**	**AAU87042**	**5.28/29.8**	**253**	**36**	**NA**	**2.0**	**>0.05**
25	**Cold inducible RNA-binding protein ( *S. salar *)**	**ADM16300**	**5.46/16.5**	**391**	**33**	**GO383013**	**2.9**	**0.0029**
26	FK-506 binding protein 1A (*G. morhua*)	AEK21706	6.71/11.8	154	32	NA	<1.5	>0.05
29	Proteasome activator complex subunit 1 (*S. salar*)	ACM09791	5.54/28.62	473	18	GW854458	<1.5	>0.05
30	PREDICTED: Calreticulin-like isform 2 (*O. niloticus*)	XP_003448811	4.43/47.146	325	41	ES771164	<1.5	>0.05

N-spot number, Acc. No.-Accession number, pI-isoelectric point, *MW*, Molecular weight; *IS*, Ion score; *SC*, Sequence coverage; *FC*, Fold change; *NA*, Not applicable.

Protein spots were identified by LC-MSMS. Proteins either with p < 0.05 or fold change >1.5 or both are in bold letters.

The rather low number of significantly different protein spots identified in this study could be linked to the possible variation in the progression of *V. anguillarum* infection in individual fish. This could be a disadvantage while analyzing samples from naturally infected fish. Besides, comparative 2D gel analysis has its limitations with regard to high abundant proteins being overrepresented in the gels, which can mask the detection of low abundant inflammatory molecules like interleukins. Also *V. anguillarum* has been shown to have high levels (up to 70 fold) of protease activity in the presence of gastrointestinal mucus [24]. This coupled with the fact that protease inhibitors were not used in the present study might have resulted in degradation of host proteins thereby affecting their detection. Nevertheless, the present approach helps in understanding the involvement of protein components during the natural progression of vibriosis in cod.

**Figure 3:**
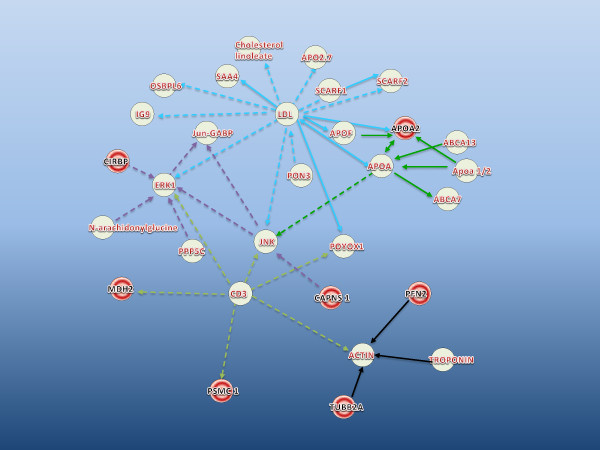
**Ingenuity pathway analysis of some key proteins identified in the study. Each gene involved in the pathway is denoted by their ENTREZ gene symbol or in some cases full gene name. **The proteins indicated in red circles showed statistically significant (p < 0.05) differential expression and/ or fold change >1.5 in comparative 2D gel analysis. Grey circles indicate other proteins involved in the pathway. Different coloured arrows are shown to highlight the central molecules involved in the pathway namely; CD3, JNK, ERK1, LDL and APOA. Solid arrows indicate direct protein interactions and dashed arrows indicate indirect protein interactions. Pathway analysis was done based on the mammalian orthologs of the proteins identified in cod mucus.

Proteomic studies to elucidate host responses in fish infected with *V. anguillarum* are limited. To gain a better understanding of the possible role of identified proteins in disease progression and inflammation, a protein-protein interaction network generated by Ingenuity Pathway Analysis (IPA) is shown in Figure 3. The network highlights the interaction of lipoproteins (LDL), mitogen activated protein kinases (ERK1, c-Jun N-terminal kinases (JNK)) and CD3 with proteins identified in the study. The afore mentioned protein families especially the JNKs are activated by stress and inflammatory signals [25] and might have been triggered by a combination of temperature/handling stress the fish had been subjected to, followed by the *V. anguillarum* infection.

Calpain small subunit 1 (CAPNS1) was found to be downregulated in the present study (spot number 9, Figure 2). CAPNS1 is a regulatory component of the calcium dependent cysteine proteases and is ubiquitous in mammalian cells [26]. Calpain mediated proteolysis is limited and calpain usually modifies/alters the substrate proteins instead of bringing about complete degradation [27] indicating its ability to specifically trigger downstream physiological response. There is also evidence that inflammatory cytokines like TNF-α and interleukins can downregulate calpain in cells and tissues [28]. Upregulation of IL-1β and IL-8 has been observed in the gut of Atlantic cod after intraperitoneal injection with live *V. anguillarum*[29]. Similarly, upregulation of these genes in cod gut were reported after immersion challenge with *V. anguillarum*[30]. Therefore it is likely that inflammatory cytokines induced during *V. anguillarum* infection in cod might have resulted in low levels of CAPNS1 observed in the present study (Figure 2). As *V. anguillarum* infections are characterized by hemorrhagic lesions and wounds in the skin, the downregulation of calpain is logical as the presence of active proteases might lead to exacerbation of disease and irreversible tissue damage [31]. Besides, epidermal lesions that may cause calcium imbalance and disturb membrane integrity can affect the activation of calpain. Human calpain II has been found to be upregulated in psoriasis skin lesions, which are characterized by calcium deficiency [31]. Calpain has been reported in different tissues of fish [32]. Mucosal specific calpains present in higher vertebrates [26] are thought to play important roles in mucosal defense.

Cold inducible RNA binding proteins (CIRBP) are stress proteins with RNA binding domain that can regulate cellular activity under stressful conditions such as hypoxia [33] or during brief periods of osmotic stress [34] in fish. In the present study, *Cirbp* was significantlyup regulated in infected fish (spot number 25, Figure 2. CIRBP influence the MAPK pathway leading to downstream inflammatory responses as shown in Figure 3.

Malate dehydrogenase 2 (mitochondrial) (MDH2) is a mitochondrial isozyme of malate dehydrogenase that is primarily involved in citric acid cycle [35]. Conditions that alter metabolism can result in elevation of mitochondrial enzymes like malate dehydrogenase [36]. An upregulation of MDH2 was noted in the infected cod (spot number 13, Figure 2), possibly triggered by *V. anguillarum* lipopolysacharide (LPS) as shown in a comparative proteome study of *Moraxella* sp. infected kidney in sea bream (*Sparus aurata*) [37].

Keratin is a cytoskeletal protein whose primary function is to protect cells from mechanical and non mechanical injuries [38]. Recent reports have also shown that keratin from fish mucus possesses antibacterial activity owing to its pore-forming properties [39]. Keratin turnover is dependent on ubiquitin-proteasome pathway and its expression levels can be altered upon cell injury [40]. There was a downregulation of Keratin II in cod mucus following vibrio infection (spot number 18, Figure 2). Beta-2-tubulins (TUBB2) form integral components of cytoskeleton and are building blocks of microtubules [41]. *Tubb2* downregulation has been reported in Chinese white shrimp (*Fenneropenaeus chinensis*) challenged with *V. anguillarum*[42]. Being an important component of cytoskeletal architecture, different expression levels of tubulin can be presumed to influence the formation of new phagosomes and thus affect phagocytic activity. Whether these changes could be pathogen mediated needs to be investigated. In the present study infected fish showed an increase in TUBB2 in the skin mucus (spot number 23, Figure 2), possibly due to increased expression of *Tubb2* in the mucosal cells associated with phagocytic processes.

14-kDa apolipoprotein (apo-14kDa) is a fish specific high density lipoprotein (HDL) orthologous to mammalian apolipoproteinA-II (apoA-II) [43], the expression of which was significantly increased in infected fish (spot number 19, Figure 2). In carp (*Cyprinus carpio*) it has been shown to have antibacterial effects *in vitro*[44]. Further, apo-14kDa was upregulated in the liver of *V. anguillarum* infected European seabass (*Dicentrarchus labrax*) [45].

Glutathione-S-transferase omega-1 (GSTO1) is an enzyme involved in biotransformation of compounds including toxic substances and oxidative stress products, transport of ligands, and regulation of signaling pathways [46]. In cod, the *Gsto1* transcripts were found in different tissues including skin [47]. In the present study, the protein amount was reduced in infected fish (spot number 12, Figure 2). In Manila clam (*Venerupis philippinarum*) infected with *V. anguillarum*[48] and Chinese mitten crab (*Eriocheir sinensis*) injected with *Aeromonas hydrophila*, an upregulation of novel *gst* transcripts was noted as early as 6 h post infection [49]. The upregulation of *gst* reported in the aforementioned studies could be due to reactive oxygen metabolites induced by the *V. anguillarum* infection as shown in a study where the measurement on enzyme activity complemented the gene expression data [50].

Proteasome 26 subunit is involved in ATP dependent degradation of ubiquitinated proteins, which are instrumental in oxidative stress response and partakes in generating peptide fragments of invading pathogen for antigen presentation [51]. Proteasome expression changes have been reported in response to *V. anguillarum* infection [42] and in Atlantic cod challenged with formalin killed *A. salmonicida*[52]. In the current study, Proteasome 26S levels were significantly higher in the infected fish (spot number 14, Figure 2).

Other important proteins that exhibited a differential expression trend in this study, including profilin-2, flotilin-1, peroxiredoxin-6, apolipoprotein AI are also important from an immune perspective. The genes corresponding to these proteins could also be considered as potential biomarkers in studies involving *V. anguillarum* infection.

In addition to bacterial infection, Atlantic cod may be susceptible to concurrent or secondary infections with parasites like *Trichodina* spp [53]. Attempts to identify ectoparasites in skin or gills was not done, hence teoretically such infections may be present and also trigger differential expression of certain proteins in the skin of cod.

### Gene expression studies on skin from experimentally challenged fish

In order to understand if the changes in the mucus proteins, arising from natural infection, correspond to the alterations of the respective genes in skin, a controlled bath challenge experiment was performed. The observations were made only during early stages of infection. The *V. anguillarum* H610 challenge protocol at Institute of Marine Research (IMR), Bergen is robust and well established as reported in different studies [7,54].

**Figure 4:**
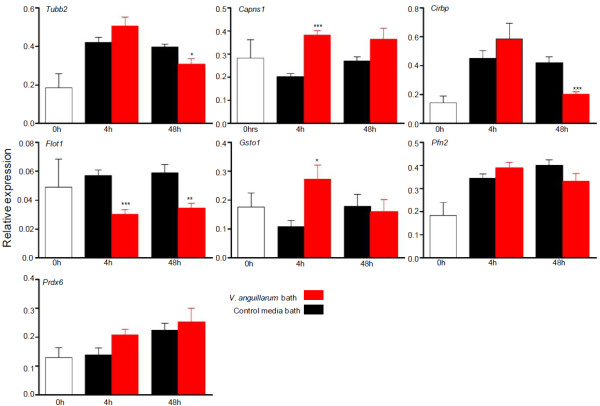
**Relative expression levels of genes (based on the proteome study) in Atlantic cod skin upon *V. anguillarum *bath challenge. **Quantification by real-time PCR. Values are indicated as means ± SEM (N = 5). Transcript levels normalized to reference genes: Ubiquitin (*Ubi*) and acidic ribosomal protein (*Arp*). Asterisk (*p < 0.05 or **p < 0.001 or ***p < 0.0001) above the error bars indicate statistically significant difference (Student’s t-test) between infected and control fish, at each time point. Initial samples are depicted as 0h samples. Standard gene abbreviations are used.

Since the proteins were identified through ESTs, the genes had to be cloned. The qPCR expression patterns of selected genes are shown in Figure 4. Significant differential expression of mRNA was detected as early as 4 h post infection. *Capns1* and *Gsto1* were upregulated at 4 h post challenge whereas *Flot1* was significantly downregulated compared to the mock challenged controls at 4 h and 48 h post infection (Figure 4). Expression of *Cirbp* and *Tubb2* were significantly downregulated 48 h post infection (Figure 4). There was no significant difference in the expression patterns of *Pfn2* and *Prdx6* (Figure 4). To confirm infection after experimental bacterial challenge, interleukin-1β (*il1β*) transcripts were quantified in the skin and gill tissue of the infected fish. The *il1β* mRNA levels were found to be significantly upregulated in gills at 48 h post bath challenge compared to the control [see Additional file 1]. *il1β* transcripts of skin showed no changes at the two time points (data not shown).

The gene expression data and proteomic data need not always show a one to one correspondence [55]. Nevertheless, as observed in the qPCR study these gene transcripts show a differential expression during the early stages of *V. anguillarum* infection and it can be surmised that they may play important role in the progression of vibriosis in cod. Fish skin has been seldom targeted for gene expression studies, in challenge experiments and infection scenarios, as most of them focused on systemic immune-relevant organs like spleen and head kidney. The expression of key immune genes in skin has been explored primarily in the case of ectoparasite infections [56]. A recent report on the expression of key immune relevant genes in naive Atlantic cod skin [57] taken together with the present study demonstrates the role of immune relevant components in the skin mucus/skin of cod both at protein and transcript level during bacterial infection.

Thus, through the proteomic approach we were able to identify differentially regulated proteins in skin mucus that might be relevant targets for gene expression studies as evident from this study. Going further, the ultimate objective of this study is to utilize the mucosal proteins as biomarkers for early assessment of fish health status, aimed at controlling bacterial diseases including vibriosis.

## Conclusions

We identified a group of proteins, of which the involvement of some can be considered novel in *V. anguillarum* infection of cod. More importantly, these proteins are shown to be closely associated with key pathways that mount an immune response in the host. The early induction of mRNA transcripts of some of the selected genes also points towards their involvement along with other acute phase proteins as primary orchestrators in the fish mucosal immunity against bacterial infection.

## Competing interests

The authors declare that they have no competing interests.

## Authors' contributions

Conceived and designed the experiments: BR, VK and MFB. Performed the experiments: BR and LJ. Analyzed the data: BR and MFB. Wrote the paper: BR, VK and MFB. All authors read and approved the final manuscript.

## Supplementary Material

Additional file 1 **Relative expression of interleukin-1β **(***il1β***) **in gill tissue of Atlantic cod bath challenged with *Vibrio anguillarum. ***Quantification of *il1β *mRNA level by real-time PCR at 4 h and 48 h post bath challenge. Values are indicated as means ± SEM (N = 5). Transcript levels normalized to reference genes: Ubiquitin (*Ubi*) and acidic ribosomal protein (*Arp*). Asterisk (*p < 0.05) above the error bars indicate statistically significant difference (Student’s t-test) between infected and control fish, at each time point. Click here for file
