# Supplementary material for: Differentially expressed proteins in the skin mucus of Atlantic cod (Gadus morhua) upon natural infection with Vibrio anguillarum
Source: BMC Vet Res. 2013 May 14;9:103. doi: 10.1186/1746-6148-9-103 (PMC3666997; doi:10.1186/1746-6148-9-103)
Supplement: Additional file 1 — Relative expression of interleukin-1β (il1β) in gill tissue of Atlantic cod bath challenged with Vibrio anguillarum. Quantification of il1β mRNA level by real-time PCR at 4 h and 48 h post bath challenge. Values are indicated as means ± SEM (N = 5). Transcript levels normalized to reference genes: Ubiquitin (Ubi) and acidic ribosomal protein (Arp). Asterisk (*p < 0.05) above the error bars indicate statistically significant difference (Student’s t-test) between infected and control fish, at each time point. [file 1746-6148-9-103-S1.pptx]

## Slide 1
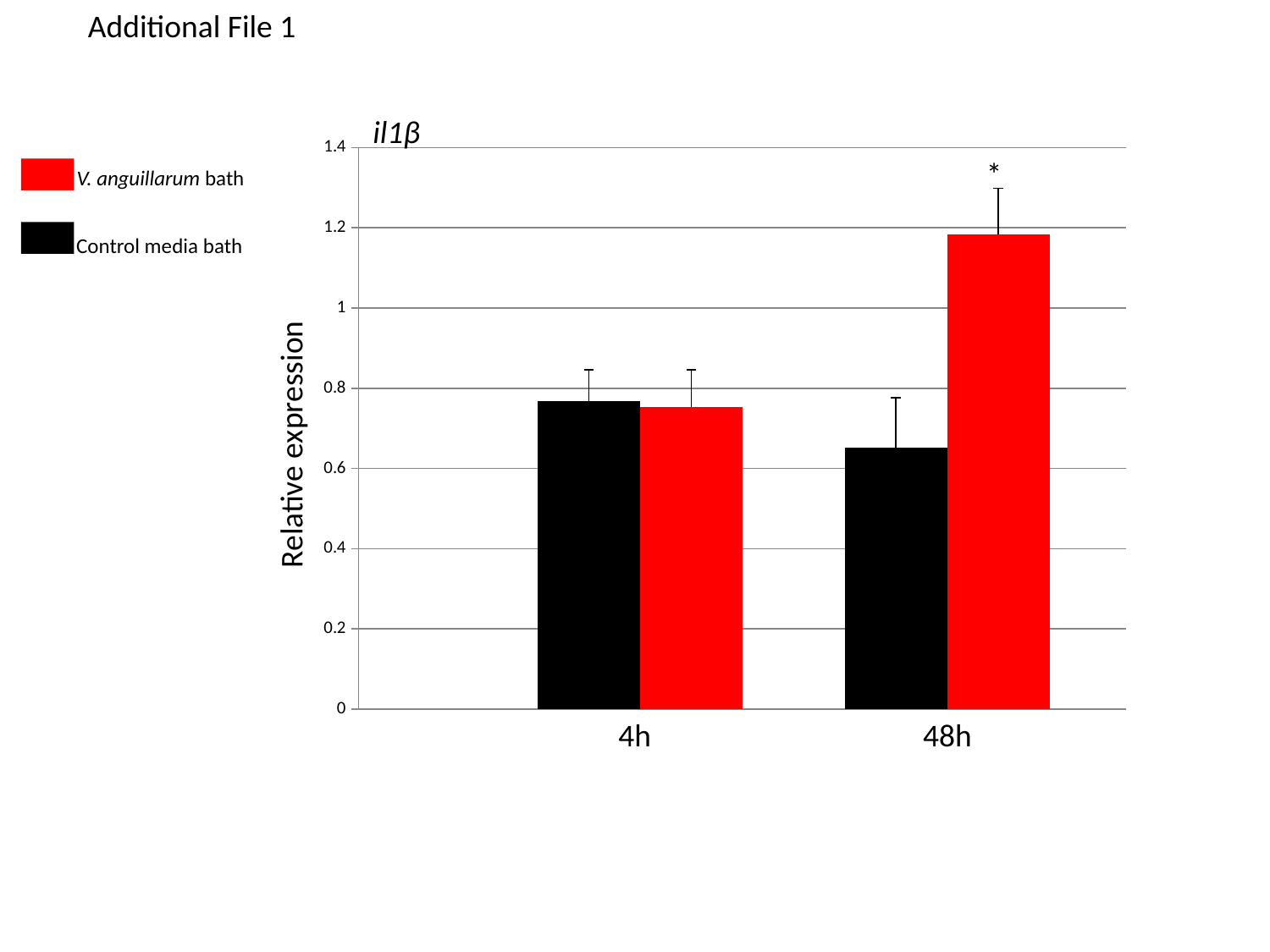

Additional File 1
il1β
### Chart
| Category | | | | | | |
|---|---|---|---|---|---|---|*
V. anguillarum bath
Control media bath
Relative expression
4h
48h
